# Static vs. Dynamic Acute Stretching Effect on Quadriceps Muscle Activity during Soccer Instep Kicking

**DOI:** 10.2478/hukin-2013-0066

**Published:** 2013-12-31

**Authors:** Mohammadtaghi Amiri-Khorasani, Eleftherios Kellis

**Affiliations:** 1 Department of Sports Biomechanics, Faculty of Physical Education and Sports Science, Shahid Bahonar University of Kerman, Kerman, Iran.; 2 Laboratory of Neuromechanics, Department of Physical Education and Sport Sciences at Serres, Aristotle University of Thessaloniki, Greece.

**Keywords:** Muscle activation, Quadriceps, Kicking velocity, Warm-up

## Abstract

The purpose of this study was to compare the effects of static and dynamic stretching on quadriceps muscle activation during maximal soccer instep kicking. The kicking motion of twelve male college soccer players (body height: 174.66 ± 5.01 cm; body mass: 72.83 ± 4.83 kg; age: 18.83 ± 0.75 years) was captured using six synchronized high-speed infra-red cameras whilst electromyography (EMG) signals from vastus medialis (VM), lateralis (VL) and rectus femoris (RF) were recorded before and after static or dynamic stretching. Analysis of variance designs showed a higher increase in knee extension angular velocity (9.65% vs. −1.45%, p < 0.001), RF (37.5% vs. −8.33%, p < 0.001), VM (12% vs. −12%, p < 0.018), and VL EMG activity (20% vs. −6.67%, p < 0.001) after dynamic stretching exercises. Based on these results, it could be suggested that dynamic stretching is probably more effective in increasing quadriceps muscle activity and knee extension angular velocity during the final swing phase of a maximal soccer instep kick than static stretching.

## Introduction

Pre-exercise warm-up routines are common practice for soccer players. Although there are various types of exercises that can be used for stretching, the advantages and disadvantages of each method have not yet been clarified (Amiri-Khorasani et al., 2011a, 2010a,c; Needham et al., 2009; Lees and Nolan, 1998). For this reason, warm-up protocols tend to reflect the experience or personal view of individual soccer coaches, trainers, and athletes.

Traditionally, static stretching exercises have been a prominent feature of warm-up routines (Power et al., 2004). However, evidence suggests that static stretching decreases force production due to, first, an alteration in length-tension relationship and, second, an altered reflex sensitivity which reduces muscle activation (Amiri-Khorasani et al., 2011a, 2010a,c; Power et al., 2004; Cramer et al., 2004; Nelson et al., 2001a,b). Acute static stretching impairs performance in vertical jumps, short sprints, tasks requiring maximal voluntary contractions, muscle strength and endurance as well as balance tasks (Amiri-Khorasani et al., 2011a, 2010a,c; Cramer et al., 2005, 2004; Behm et al., 2001; Church et al., 2001). Therefore, the benefits of static stretching for performance seem questionable.

Several research studies have emphasized the positive effects of dynamic stretching for performance as opposed to static stretching (Amiri-Khorasani et al., 2011a, 2010a,c; Power et al., 2004; Cramer et al., 2004; Nelson et al., 2001a,b). Particularly, it has been suggested that dynamic stretching increases force production as a result of post-activation potentiation (PAP) and a higher muscle temperature (Herda et al., 2008). PAP is caused by a voluntary conditioning contraction which is executed in a consistent manner at a maximum or near maximum intensity (Faigenbaum et al., 2005; Yamaguchi and Ishii, 2005; Behm et al., 2001). PAP enhances peak isometric force and the rate of force development during twitch contractions (Tillin and Bishop, 2009; Docherty and Hodgson, 2007; Hodgson et al., 2005; Robbins, 2005; Sale, 2002) perhaps by an altered phosphorylation of myosin regulatory light chains (Baudray and Duchateu, 2007; Sale, 2004; Chiu et al., 2003; Hamada et al., 2003, 2000; Gossen and Sale, 2000) and more recruitment of higher order motor units (Hodgson et al., 2005; Chiu et al., 2003). Tillin and Bishop (2009) suggested that PAP is able to potentially increase mechanical power as well as explosive activity and hence, performance and/or the training stimulus of that activity. To our knowledge, the effect of dynamic stretching on performance of a multiarticular sport skill, such as the soccer kick, has not been thoroughly investigated.

The instep kick is one of the most characteristic skills of a soccer player (Amiri-Khorasani et al., 2011a,b, 2010a,b, 2009; Kellis and Katis, 2007; Kellis et al., 2006). Soccer kicks performed via a stretch-shortening cycle of the knee extensors display higher ball velocity compared to soccer kicks involving only concentric actions (Bober et al., 1987). For this reason, research studies have focused on the role of stretch-shortening cycle of the knee extensors for a successful kick. Particularly, a soccer kick is accompanied by a stretch of the knee extensor musculature during backswing followed by instantaneous shortening during forward shank movement. Each phase, however, is accompanied by different behavior of the quadriceps components. During backswing, the thigh accelerates via a high activation of rectus femoris (Dorge et al., 1999; Sorensen et al., 1996). Forward swing is characterized by a high activation of vastus lateralis which then decreases when the shank starts to decelerate (Dorge et al., 1999; Sorensen et al., 1996). It seems, therefore, that evaluation of only one component of the quadriceps muscle cannot fully describe the role of the whole muscle group during the soccer kick.

In previous experiments, Amiri-Khorasani et al. (2010a) reported that maximum ball speed and vastus medialis electromyographic (EMG) activation during soccer instep kicking increased more after dynamic rather than static stretching. However, in this study the experimental groupconsisted only of six subjects and EMG activation of only vastus medialis was analyzed. It is not clear whether this reflects the whole quadriceps muscle or the effects of stretching are muscle-dependent. Since some of the quadriceps are mono-articular (vastii muscles) while others are bi-articular (rectus femoris) then it would be interesting to examine whether dynamic stretching affects activation of only some parts of the quadriceps and whether this differs from static stretching effects. Therefore, the purpose of the present study was to investigate the acute effects of dynamic and static stretching on vastus medialis (VM), vastus lateralis (VL) and rectus femoris (RF) activation during maximal instep soccer kicks. We hypothesized that dynamic stretching would cause a higher increase in quadriceps muscle activation compared to that observed after static stretching.

## Material and Methods

### Participants

Twelve male college soccer players (mean ± SD: body height: 180.08 ± 4.16 cm; body mass: 78.16 ± 4.44 kg; age: 19.16 ± 0.83 years), who had no history of major lower limb injury or disease, volunteered to participate in this study in the middle of the 2010–2011 season, after providing their informed consent. The University Ethics Committee gave approval for all procedures. Subjects were required to report to our research laboratory and also complete a medical questionnaire. As all participants preferred to kick the ball using their right leg, the right leg was considered for further analysis.

### Procedures

Each subject visited the laboratory twice, on separate days. In the first day, the subject performed one (out of the two) stretching protocols. Soccer kick kinematics and muscle EMG were recorded before and after each protocol. The protocol plan included jogging for 4 minutes, 5 soccer instep kicks, performing stretching routines, 2 minutes rest and eventually 5 soccer instep kicks respectively (Table 1). The whole procedure was repeated on the second day, but the order of stretching protocols was reversed. The sequence of stretching protocol performance was randomized across subjects and days such that half of the subjects received first static and then dynamic stretching and the other half received the reverse sequence. The reason for applying this specific design was that we could control error factors, such as effects of testing, weather, pitch and time of the day.

Six synchronized high-speed infra-red cameras (Vicon MX-F20, Oxford Metrics Ltd., Oxford, UK) were used to capture limb motion at 200 Hz. Once the cameras were positioned into the appropriate locations (performance area), then they were calibrated to define their own volume origin. A motion capture software (Vicon Nexus 1.2, Oxford Metrics Ltd., Oxford, UK) was used to digitize body landmarks, including the bony anatomical landmarks of the right and left anterior superior iliac spine, posterior superior iliac spine, mid-way between the posterior superior iliac spines, lateral epicondyle of knee, thigh; over the lower lateral ⅓ surface of the thigh, lateral malleolus, shank; over the lower ⅓ of the shank, over the fifth metatarsal head, and calcaneous. A static calibration trial was performed prior to dynamic data collection to detect marker placement errors if/when necessary. In this trial, subjects were asked to stand in anatomical position in the center of the capture volume.

EMG data were collected on-line at 1200 Hz with a TeleMyo telemetric hardware system (Noraxon, USA, Inc., Scottsdale, AZ) with a 16-bit A/D board (National Instruments, Austin, TX) of the motion capture system. Particularly, surface EMG signals from the swinging leg (1) rectus femoris (RF), half way between the anterior superior iliac spine (ASIS) and the superior border of the patella; (2) vastus medialis (VM), 20 % of the distance from the medial knee joint line to the ASIS; and (3) vastus lateralis (VL), 25 % of the distance from the lateral joint line to the ASIS were recorded from pre-gelled silver-silver/chloride bipolar surface electrodes (Medicotest A/S, Rugmaken, Denmark). These muscles were selected because they demonstrate high activity during a soccer kick (Manolopoulos et al., 2004; Dorge et al., 1999; DeProft et al., 1988). The participant's skin was prepared to reduce skin impedance before placement of EMG electrodes. Hair at the site of electrode placement was removed by shaving, and skin was abraded with an abrasive pad and cleaned with isopropyl alcohol. Then, the electrodes were placed over each muscle belly in line with the direction of the fibers with a center to center distance of approximately 2.5 cm. A single ground electrode was placed over a lateral femoral condyle.

#### Stretching programs

**Figure 1:**
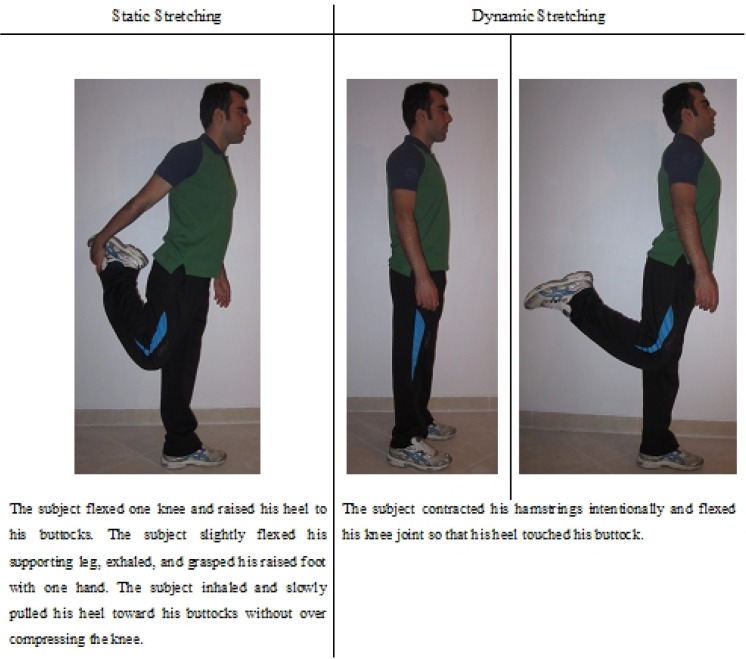
Static and dynamic stretching of the quadriceps

The quadriceps muscle group was stretched via a static and dynamic stretching program, previously explained by Little and Williams (2006) and Amiri-Khorasani et al. (2010a,c and 2011a). For static stretching, as shown in Figure 1, subjects held the stretch for 30 seconds on one leg before changing to the contralateral side. Subjects were instructed to stretch gradually to the end of the range of motion (ROM) while ensuring that motion was well within the pain threshold for injury.

The procedures for performing dynamic stretching on the quadriceps (Figure 1) have been previously explained in detail (Amiri-Khorasani et al., 2011a, 2010a,c; Yamaguchi and Ishii, 2005). From the standing position, each subject intentionally contracted the antagonist muscle (hamstring) so that the target (quadriceps) muscle was stretched. They were instructed to achieve maximal ROM during each repetition. Five stretches were performed at three different speed conditions: slow, moderate and “as fast as possible”, thus leading to an overall number of 15 stretches, each lasting 1 s. The order of exercises and resting periods was the same as those in the static stretching.

#### Instep Soccer kicks

A ball was kicked 3 m towards a target 1 × 1 m in size. A FIFA-approved size five soccer ball (mass = 0.435 g) was used for each kicking session and its inflation was controlled throughout the trials at 700 hPa. To minimize movement in the frontal plane, the participants were restricted to a 3 m straight run-up from a position directly behind the ball at an approach angle of 0°. Five kicks, as hard as possible, were performed and the kick which displayed the highest ball velocity against the target was further analyzed.

#### Data analysis

The three-dimensional coordinates were expressed in a global right-handed orthogonal reference frame whose origin was placed at the ground level, with the Y-axis pointing toward the direction of the ball, the Z-axis vertically upward and the X-axis perpendicular to X and Y. The threedimensional knee extension–flexion, hip extension–flexion and ankle plantarflexion–dorsiflexion angles were then estimated and used for analysis. For reference, 180 deg indicated full knee extension and normal standing position, respectively. The ankle in a neutral position was equal to 90 deg (angles 0–90 deg indicated dorsiflexion and angles 90–180 deg indicated plantarflexion).

The raw EMG data were low-pass filtered at 500 Hz and high-pass filtered at 10 Hz to eliminate movement artefacts, using a Butterworth fourth-order zero-lag filter. The onset/offset time selected from starting knee extension of the swinging leg to impact the ball. After removing the signal offset, the root mean square (RMS) was estimated from raw EMG signal data using a smoothing window.

In each kick, we examined the (1) maximum RMS of RF, VM and VL muscles, (2) maximum knee angular velocity (KAV), (3) maximum ankle angular velocity (AAV), (4) maximum foot velocity (FV) and (4) maximum ball velocity (BV). Foot velocity (Vfoot) was estimated as the velocity of the center of mass of the foot, which was calculated in each frame based on ankle and toe marker data. The mechanics of collision between the foot and ball were analyzed as suggested by Lees and Nolan (1998). Particularly, the resultant ball velocity (Vball) was calculated from V foot as follows: v_ball_ = 1.23 × v_foot_ + 2.72

The Pre-stretching and Post-stretching values for each protocol were averaged across days and therefore for each participant there were four values: pre- and post- static stretching and pre- and post-dynamic stretching ones. Subsequently, in each variable, the percentage differences between pre- and post- stretching protocol were calculated and compared between protocols.

### Statistical Analysis

A one-way analysis of variance was used to compare relative changes in each dependent variable between static and dynamic stretching. The level of significance was set at p ≤ 0.05. When justified, paired sample t-tests were performed to confirm significant changes within each condition. Effect sizes (ES) were calculated and are also reported. The power was ≥ 0.94 and the test–retest reliability values for the testing order of tests ICCRs (intraclass correlation reliability) were ≥ 0.97.

## Results

**Figure 2:**
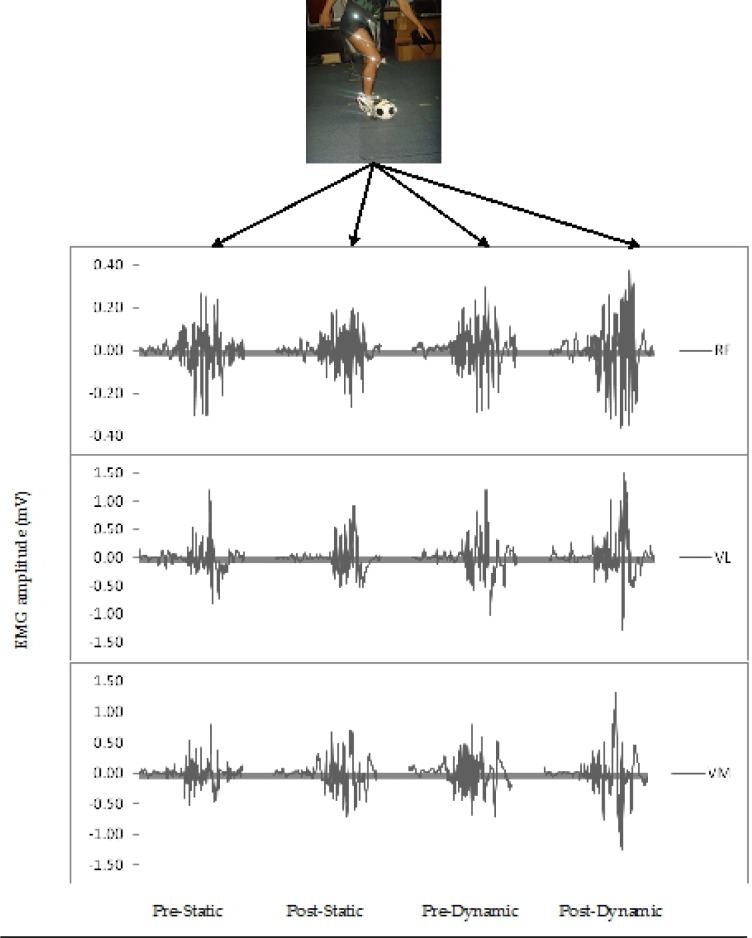
Example of raw EMG of rectus femoris (RF), vastus lateralis (VL), and vastus medialis (VM) after different acute stretching methods (pre-static, post-static, pre-dynamic, and post-dynamic) during soccer instep kicking

**Figure 3:**
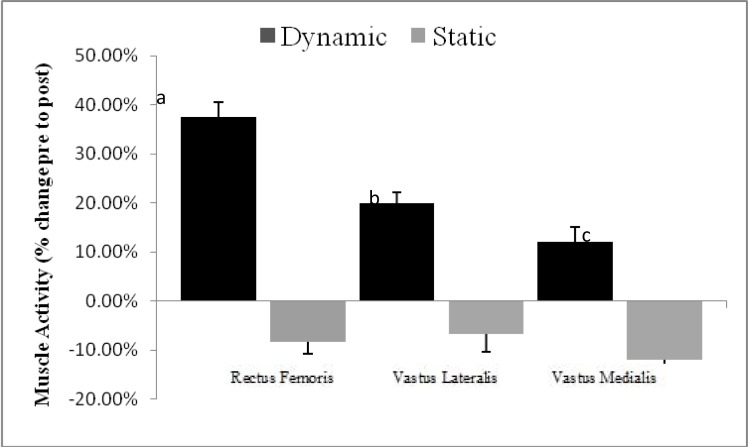
*Mean ± SD changes in rectus femoris, vastus lateralis, and vastus medialis root mean square EMG during soccer instep kicking before and after static and dynamic stretching.*
Significant at p < 0.015,Significant at p < 0.004,Significant at p < 0.049

An example of EMG raw data of RF, VL, and VM activity after different acute stretching methods is illustrated in Figure 2. The descriptive results of raw EMG and KAV data are presented in Table 2 while mean group values are presented in Figure 3. The ANOVA showed a statistically significant higher increase in RF EMG (Figure 3) after dynamic stretching (37.50% ± 9.37%) versus a non-significant (−8.33% ± 3.89%) decrease after static stretching (p = 0.015) (ES ≥ 0.94). Similarly, VL EMG increased after dynamic stretching (20% ± 10.21%) but it decreased (−6.60% ± 8.77%) after static stretching (p = 0.004) (ES ≥ 0.98). There was also a statistically significant increase in VM EMG after dynamic stretching (12.00% ± 6.29%) as opposed to a decrease (−12.00% ± 5.64%) after static stretching (p = 0.049) (ES ≥ 0.97).

KAV showed a significant increase by 9.65% ± 4.92% after dynamic stretching (p = 0.002) versus a non-significant change (−1.45% ± 4.84%) after static stretching (ES ≥ 0.98). Dynamic stretching (10.12% ± 5.32%) also showed greater AAV than static stretching (−3.29% ± 3.68%) (p = 0.011) (ES ≥ 0.96). In addition, dynamic stretching (10.77% ± 7.12%) caused significantly faster BV when compared to static stretching (−6.56% ± 3.67%) (p = 0.001) (ES ≥ 0.99).

## Discussion

The main finding of this study is that, compared to static stretching, dynamic stretching of the quadriceps resulted in a higher increase of (1) VM, VL and RF muscle activation, (2) maximum knee and ankle angular velocity and (3) maximum ball velocity during an instep soccer kick. Further, dynamic stretching caused a higher increase of RF muscle activity as opposed to VM and VL muscles.

The present results support previous research studies (Cramer et al., 2005; Marek et al., 2005) indicating that dynamic stretching increases activation of all superficial quadriceps muscles more than static stretching (Figure 3). However, in contrast to previous research studies, our results refer to a multiarticular movement, such as the soccer kick and therefore, direct comparison between the aforementioned studies is difficult. Particularly, backward and forward swinging motion of the kicking leg is mainly accompanied by a fast stretch-shortening cycle of the quadriceps (Bober et al., 1987). Along with the motion-dependent moments, the knee extensors provide the main force in order to accelerate the shank during the forward motion of the kicking leg (Kellis et al., 2006; Dorge et al., 1999).

A higher quadriceps activation and strength, coupled with a more efficient stretch-shortening cycle probably lead to a higher maximal KAV (Kellis and Katis, 2007; Kellis et al., 2006) which is transmitted to the ankle and finally to the toe and increases ball speed (Asami and Nolte, 1983). Consequently, any changes observed after stretching should be related to some or all the aforementioned factors.

In the present study, quadriceps muscle EMG (Figure 3) remained unaltered while angular and ball speed kinematics decreased after static stretching. Therefore, static stretching had an overall negative effect on soccer kick performance. Previous studies reported that static stretching reduced quadriceps EMG activity and maximum peak torque during maximum strength testing (Marek et al., 2005; Cramer et al., 2005; Behm et al., 2001) and soccer kicking (Amiri-Khorasani et al., 2010a). This provides an initial explanation of the reduction of ball speed after static stretching. Neural factors which include alterations in Golgi tendon organ reflex activity, mechanoreceptor and receptor pain feedback, and/or fatigue related mechanisms (Fowles et al., 2000) may have contributed to the maintenance of quadriceps EMG after static stretching. Others proposed that it is a result of temporary impairment of gamma loop role (Herda et al., 2008) or a CNS response to stretching (Cramer et al., 2005). In addition, static stretching might cause an increase in the compliance of the muscular tendon unit (MTU) (Amiri-Khorasani et al., 2010b; Herda et al., 2008; Fowles et al., 2000) which has been hypothesized to alter the force-relaxation properties within a muscle, thereby decreasing its force-generating capacity (Kokkonen et al., 1998; Rosenbaum and Hennig, 1995; Wilson et al., 1994). The higher compliance may be attributed to changes in tendon compliance (Kubo et al., 2001), fascicle length (Fowles et al., 2000), and intramuscular connective tissue elasticity (Morse et al., 2008). In fact, Herda et al. (2008) suggested that higher stiffness increases muscle force production and activation and it finally produces more angular velocity around the joint, which was absent after our static stretching protocol.

In contrast to static stretching, dynamic stretching showed a higher activation of quadriceps which probably increased KAV during the soccer kick. This could be attributed to several factors. First, the increase in quadriceps activation might have also increased the stiffness of the MTU, thus increasing maximum force production of these muscles during the kick (Herda et al., 2008).

A stiffer MTU may also allow a better energy transfer during the stretch-shortening cycle of the quadriceps during the kick. Second, dynamic stretching increases force production and muscle activation as a result of PAP and, perhaps, a higher muscle temperature (Herda et al., 2008; Wilson et al., 1994). In this case, PAP is able to increase mechanical power and explosive activity and, hence, performance (Tillin and Bishop, 2009). This effect might be more evident in the soccer kicking movement, which is highly explosive. Third, in our study, the participants were asked to perform five slow, five moderate, and five rapid quadriceps stretching exercises. Such a stimulus has been shown to enhance neuromuscular propagation perhaps by increasing the number of active motor units (Hicks et al., 1989).

It was also interesting that dynamic stretching improved angular velocity of the ankle during the kick (Table 2). Since the quadriceps muscle is not activated around the ankle, the exact reason for this finding is not clear. We can assume that two factors are important: first, that stretching the quadriceps enhances energy transfer from the proximal to the distal segments, thus, increasing the velocity of the end-point segment (Kellis et al., 2006) and, second, there may be a possibility that whilst subjects stretched their quadriceps a certain amount of ankle muscle stretching took place.

Another interesting finding of this study was that dynamic stretching had a much higher effect on RF muscle as opposed to the vastii muscles, especially the VM (Figure 3). This must be linked with the bi-articular nature of the RF as opposed to the monoarticular VM and VL muscles. Particularly, the RF acts simultaneously as a hip flexor and as a knee extensor, therefore, its contribution, in terms of muscle activation, is likely to be higher as it is activated both during backswing (Dorge et al., 1999; Sorensen et al., 1996) and forward swing while the vastii muscles show higher activation mainly during the forward swing phase (Dorge et al., 1999). A higher activation state by the RF seen after dynamic stretching would indicate a stiffer RF muscle and, in turn, a more effective energy transfer through the stretch-shortening cycle of that muscle. This would also allow a better energy transfer from the hip to the knee joint of the swinging limb during the kick. This may not be the case for the vastii muscles, which are mono-articular and therefore, they may not be similarly stretched and activated during the kick.

We concluded that dynamic stretching as compared to static stretching causes higher muscle activation to perform maximum effort due to PAP. Therefore, it produces more torque around joints which causes grater angular velocity of joint and finally, high ball velocity. Hence, dynamic stretching during a warm up creates higher ball velocity by higher muscle activation. In practical application view, compared to static stretching, dynamic stretching is probably more effective for enhancing powerful soccer instep kick performance. Therefore, coaches, trainers, and physical educators should design soccer training programs by incorporating dynamic stretching as a part of the warm up. Since dynamic stretching has an acute effect on performance, the use of this type of stretching prior to games is recommended.

## Figures and Tables

**Table 2:** Mean (± SD) muscles activity, knee and ankle joints angular velocity, and foot and ball velocity descriptors of the soccer instep kicking after different acute stretching methods

**Dependent Variables**	**Pre-Static**	**Post-Static**	**Pre-Dynamic**	**Post-Dynamic**
RMS of RF (mV)	0.08 ± 0.04	0.08 ± 0.03	0.08 ± 0.02	0.11 ± 0.03
RMS of VL (mV)	0.30 ± 0.06	0.28 ± 0.06	0.30 ± 0.06	0.36 ± 0.02
RMS of VM (mV)	0.25 ± 0.07	0.22 ± 0.08	0.25 ± 0.07	0.28 ± 0.04
KAV (rad/s)	17.25 ± 1.65	17.00 ± 1.71	17.40 ± 2.36	19.08 ± 1.85
AAV (rad/s)	1.17 ± 1.08	0.99 ± 1.59	1.66 ± 0.60	1.92 ± 1.10
FV (m/s)	16.54 ± 2.43	15.34 ± 2.01	16.59 ± 1.45	18.75 ± 2.56
BV (m/s)	24.09 ± 2.16	22.51 ± 1.86	24.24 ± 1.50	26.85 ± 2.41

**Table 2:** Mean (± SD) muscles activity, knee and ankle joints angular velocity, and foot and ball velocity descriptors of the soccer instep kicking after different acute stretching methods

**Dependent Variables**	**Pre-Static**	**Post-Static**	**Pre-Dynamic**	**Post-Dynamic**
RMS of RF (mV)	0.08 ± 0.04	0.08 ± 0.03	0.08 ± 0.02	0.11 ± 0.03
RMS of VL (mV)	0.30 ± 0.06	0.28 ± 0.06	0.30 ± 0.06	0.36 ± 0.02
RMS of VM (mV)	0.25 ± 0.07	0.22 ± 0.08	0.25 ± 0.07	0.28 ± 0.04
KAV (rad/s)	17.25 ± 1.65	17.00 ± 1.71	17.40 ± 2.36	19.08 ± 1.85
AAV (rad/s)	1.17 ± 1.08	0.99 ± 1.59	1.66 ± 0.60	1.92 ± 1.10
FV (m/s)	16.54 ± 2.43	15.34 ± 2.01	16.59 ± 1.45	18.75 ± 2.56
BV (m/s)	24.09 ± 2.16	22.51 ± 1.86	24.24 ± 1.50	26.85 ± 2.41
